# Cognitive control of complex motor behavior in marmoset monkeys

**DOI:** 10.1038/s41467-019-11714-8

**Published:** 2019-08-22

**Authors:** Thomas Pomberger, Cristina Risueno-Segovia, Yasemin B. Gultekin, Deniz Dohmen, Steffen R. Hage

**Affiliations:** 1 Neurobiology of Vocal Communication, Werner Reichardt Centre for Integrative Neuroscience, University of Tübingen, Otfried-Müller-Str. 25, 72076 Tübingen, Germany; 2 Graduate School of Neural & Behavioural Sciences, International Max Planck Research School, University of Tübingen, Österberg-Str. 3, 72074 Tübingen, Germany

**Keywords:** Psychophysics, Cognitive control, Motor control

## Abstract

Marmosets have attracted significant interest in the life sciences. Similarities with human brain anatomy and physiology, such as the granular frontal cortex, as well as the development of transgenic lines and potential for transferring rodent neuroscientific techniques to small primates make them a promising neurodegenerative and neuropsychiatric model system. However, whether marmosets can exhibit complex motor tasks in highly controlled experimental designs—one of the prerequisites for investigating higher-order control mechanisms underlying cognitive motor behavior—has not been demonstrated. We show that marmosets can be trained to perform vocal behavior in response to arbitrary visual cues in controlled operant conditioning tasks. Our results emphasize the marmoset as a suitable model to study complex motor behavior and the evolution of cognitive control underlying speech.

## Abstract (web summary)

Whether marmosets can exhibit complex motor tasks in controlled experimental designs has not yet been demonstrated. Here, the authors show that marmoset monkeys can be trained to call on command in controlled operant conditioning tasks.

## Introduction

The marmoset, a small New World primate, has recently garnered considerable interest as a suitable model organism in the life sciences^1^. Similarities to humans in terms of genetic and physiological features, in combination with high fertility, short-life span, and the ease to keep them under captivity makes them an especially efficient model for biomedical research and genetics^2^. The prospect of developing primate transgenic lines^3^, their granular frontal cortex, and potential for transferring a number of rodent neuroscientific techniques to a small primate with a lissencephalic brain^1^ position the marmoset as a promising neurodegenerative and neuropsychiatric model system of prefrontal cortex dysfunctions. As an example, marmosets are highly social and vocal animals that use vocal signals for acoustic communication^4–6^, a behavior that is severely affected by neurodegenerative diseases such as Parkinson’s and Alzheimer’s disease in humans^7,8^.

To date, a variety of neurophysiological methods, as well as different brain imaging techniques have been successfully developed and established in marmosets, highlighting the potential for using these animals to study cognitive processes and their underlying neural network in different conditions and contexts^1,2^. Currently, these methods are used in anesthetized^9,10^, freely moving^11^, and restrained marmosets^12^, as well as in animals that have been trained to perform motor tasks such as licking^13,14^, saccadic eye movements^15,16^, or arm reaching^17,18^ in response to visual or auditory stimuli. However, neuroscience needs complex behaviors to learn more about certain brain–behavior relationships^19^. Unfortunately, marmosets have not yet been trained to perform complex behavioral tasks, i.e., motor output that requires several groups of muscles to be properly coordinated such as in vocal behavior, in well-controlled experimental designs, a prerequisite for the investigation of frontal neural networks underlying intricate cognitive processes, as shown in the canonical macaque model^20–22^. Therefore, providing evidence that marmoset monkeys can be successfully trained to perform complex behavioral tasks would bridge the gap and make them a suitable model system for investigating the neural network underlying cognitive processes in health and disease.

We trained four adult marmoset monkeys (*Callithrix jacchus*) to perform a computer-controlled go/no-go detection task by using their vocal behavior as a response. Vocal behavior is a complex behavior involving several groups of muscles, such as articulatory (orofacial and jaw), laryngeal and respiratory muscles, that are controlled by different motoneuron pools within the ventrolateral pontine brainstem and spinal cord that have to be coordinated in a precise timely manner to ensure proper call production^23,24^. We show that they are able to volitionally control their vocal output and use it as an immediate response to a learned, abstract visual cue, thus demonstrating the ability to instrumentalize their vocal output to perform a task successfully. Furthermore, we trained one monkey to switch between distinct call types from trial to trial in response to different visual cues in a discrimination task. Our findings show that marmoset monkeys can be trained to perform vocal behavior in a controlled experimental design suggesting their suitability as an innovative nonhuman primate model to decipher higher-order cognitive motor control mechanisms.

## Results

### Vocal performance during the detection task

**Fig. 1:**
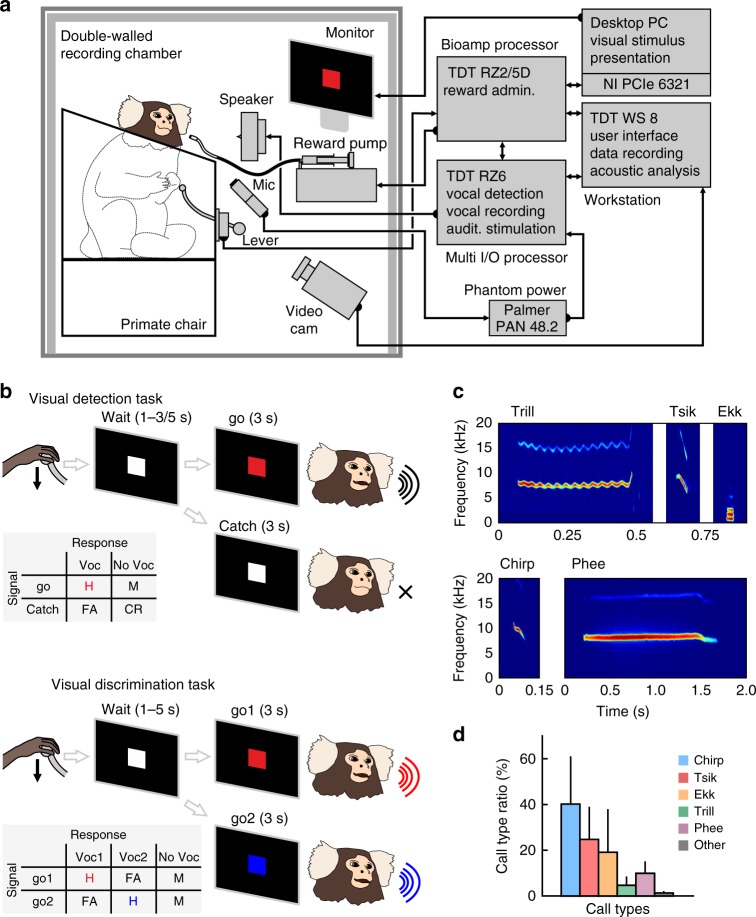
Training marmoset monkeys to call on command. **a** Experimental setup. Animals were trained in a double-walled soundproof recording chamber. The schematic block diagram depicts the system used for stimulus presentation, behavioral monitoring, and reward presentation. **b** Training paradigms. All monkeys were trained in a go/no-go protocol to vocalize whenever a red cue appeared (detection task; upper depiction). One monkey was trained in a successive training period to utter distinct vocalizations in response to red and blue visual cues, respectively (discrimination task; lower depiction). H = hit; M = miss; FA = false alarm; CR = correct rejection. **c** Spectrograms of representative vocalizations uttered by the experimental animals. **d** Mean call type distribution of the four monkeys in visual detection sessions. Whiskers indicate standard error (SE)

**Fig. 2:**
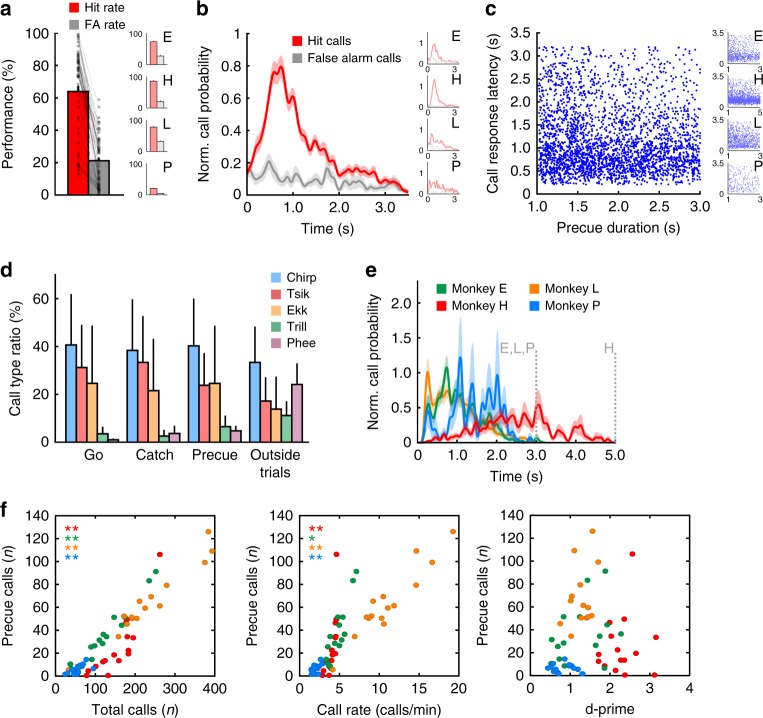
Marmosets call on command during visual detection task. **a** Mean distribution of hit and false alarm rates of 15 sessions in four animals exhibit significantly higher call probabilities during go-trials (hits) than during catch trials (false alarms). The main plot shows the group average; small plots show the mean of the four individual animals. Whiskers indicate standard error (SE). **b** Call probabilities during go-trials and catch trials. The main plot shows the group average; small plots show the mean of the four individual animals. Data were normalized for 15 sessions per animals. Shaded areas indicate SE. **c** Correlation between call response latencies after go-cue onset and the preceding waiting period (cue delay). The main plot shows the individual response latency with the corresponding cue delay for all calls uttered by the four animals in the 15 sessions, the small plots for each individual monkey. **d** Mean call type distribution for four monkeys during three time periods for self-initiated trials (pre-cue, go, and catch phase) and outside trials. Whiskers indicate standard error (SE). **e** Mean call probabilities during the pre-cue phase for the four animals. Note that monkeys E, L, and P were trained with 1–3 s and monkey H with 1–5 s pre-cue latency, respectively (gray, dashed vertical line). Shaded areas indicate SE. **f** Correlations between pre-cue calls and total number of calls, call rate (as a measure for arousal), and *d′* values for each session for each animal. Subjects are colored according to (D). *p < 0.01, **p < 0.001, Pearson’s correlation

We recorded 10,619 vocalizations from four marmoset monkeys that were uttered in 80 daily sessions while performing either a detection or discrimination task (Tables 1 and 2). In the visual detection task, monkeys were trained to sit in a monkey-chair in front of a monitor in a soundproof chamber (Fig. 1a) and required to vocalize after cueing by an arbitrary visual stimulus (red square) to receive a reward (see Fig. 1a, b for experimental setup and design and Methods for details). The data were obtained from 15 consecutive daily sessions per monkey. Within sessions, all monkeys produced a variety of different call types (Fig. 1c) with short call types such as chirps (40.6 ± 20.8%) and tsiks (25.4 ± 14.6%) occurring most frequently (Fig. 1d). All monkeys exhibited mean call counts between 55 and 232 calls/session resulting in high call rates of between 2 and 11 calls per minute and successfully performed between 72 and 174 trials per session (Table 1). Throughout self-initiated trials, all monkeys produced significantly more calls in hit than catch trials [*p* = 6.1e − 05 for each individual monkey, *n* = 15 (15 paired sessions with hit and catch-trial ratios), Wilcoxon signed-rank test]. Mean values were high for all monkeys for hit (monkey E: 72.3 ± 3.3%, monkey H: 85.2 ± 3.5%, monkey L: 77.1 ± 3.2%, monkey P: 20.8 ± 4.8%) and low for false-alarm rates (monkey E: 27.4 ± 3.5%, monkey H: 20.2 ± 4.1%, monkey L: 32.2 ± 2.8%, monkey P: 4.8 ± 0.5%), resulting in a mean population hit rate of 63.9 ± 3.6% and a mean population false-alarm rate of 21.2 ± 2.0% (Fig. 2a). These data show that the monkeys reliably produced calls in response to the visual go-cue. All monkeys showed similar and distinct response patterns during hit trials with median latencies between 828 and 1219 ms, resulting in a median population response latency of 903 ms (Fig. 2b). Next, we investigated whether the vocal response latency was dependent on the cue delay, i.e., the waiting period between self-induced trial initiation and go-cue onset. Here, a strong indirect correlation between cue delay and corresponding vocal response latency could indicate a pre-cue dependent rather than a go-cue dependent vocal onset. Therefore, we tested the relationship between the duration of the cue delay and the corresponding vocal response latency for all monkeys. Vocal response latencies did not show any clear dependency between cue delay and corresponding vocal response latency in all monkeys. While cue delays and corresponding vocal response latencies did not show any correlation in two animals (*p* = 0.18, *r* = −0.049, *n* = 736 for monkey E, and *p* = 0.88, *r* = −9.4e − 03, *n* = 255 for monkey P; Pearson’s correlation), they revealed a low yet significant indirect correlation in the other two monkeys (*p* = 8.53e − 06, *r* = −0.106, *n* = 976 for monkey L and *p* = 8.7e − 03, *r* = −0.08 *n* = 1745 for monkey H; Pearson’s correlation, Fig. 2c). However, the correlation coefficient in the latter two monkeys was close to −0.1 (as in the other two monkeys) indicating no effect of cue delays on the corresponding cue delays in these animals as well^25^. Overall, this indicates that all monkeys produced their vocalizations in response to go-onset.

**Table 1:** Vocal performance of monkeys during the visual detection task

Monkey ID	Calls in total	Calls per session	Call rate (calls/min)	Hit calls per session	Catch calls per session	Pre-cue calls per session	Trials per session
Monkey E	1845	123 ± 16	4.8 ± 0.3	53 ± 6	5 ± 1	36 ± 6	92 ± 8
Monkey H	2286	152 ± 13	4.0 ± 0.1	118 ± 7	7 ± 1	25 ± 7	174 ± 6
Monkey L	3483	232 ± 25	11.1 ± 1.0	70 ± 4	7 ± 1	63 ± 8	114 ± 6
Monkey P	822	55 ± 5	2.0 ± 0.1	18 ± 1	1 ± 0	6 ± 1	120 ± 11

**Table 2:** Vocal performance of one monkey during the visual discrimination task

Time of recording	Calls in total	Calls per session	Call rate (calls/min)	go1 calls per session	go2 calls per session	Trials per session
Pre-training	1530	153 ± 8	4.5 ± 0.1	66 ± 5	54 ± 2	160 ± 9
Post-training	653	65 ± 4	3.6 ± 0.2	24 ± 1	23 ± 1	59 ± 5

We then investigated whether animals exhibited a different ratio of call types within the three phases of the visual detection task (go, catch, and pre-cue) and outside of the self-initiated trials. Even though call type ratios differed between animals, we observed a significant difference in call type distribution between these four phases (*p* = 1.7e − 02, d*f* = 12, sum square = 2650.2, three-way ANOVA, Fig. 2d). Post hoc tests revealed that this difference could be predominantly explained by a higher occurrence of long phee calls outside of trials, which were almost completely lacking in trials (*p* = 1.9e − 02, d*f* = 3, *F* = 4.88, one-way ANOVA). Within trials, monkeys predominantly produced calls within the go phase. However, we also observed that monkeys produced a substantial number of calls during the pre-cue phase, resulting in trial abortion (Table 1, Fig. 2e). Furthermore, we measured a significant correlation between the number of such pre-cue calls and the corresponding total number of calls per session for all monkeys (*p* = 4.9e − 05, *r* = 0.86 for monkey E; *p* = 2.7e − 26, *r* = 0.93 for H; *p* = 2.7e − 10, *r* = 0.98 for L; *p* = 1.2e − 09, *r* = 0.97 for P; *n* = 15 sessions, Pearson’s correlation, Fig. 2f). We hypothesized that the number of pre-cue calls and, more generally, the corresponding total number of calls per session are directly related to the arousal state of the animal and that animals in a higher arousal state are capable of inhibiting call production to a lesser extent than monkeys in a low-arousal state. To test this, we compared the number of pre-cue calls with the corresponding sessions’ call rate, which has been shown to directly correlate with the arousal state in monkeys^26,27^. We observed a significant correlation between the number of pre-cue calls and corresponding call rate for the session, suggesting a significant role of the animals’ arousal in the overall calling behavior (*p* = 4.7e − 03, *r* = 0.86 for monkey E; *p* = 2.5e − 17, *r* = 0.84 for H; *p* = 9.5e − 06, *r* = 0.89 for L; *p* = 9.6e − 08, *r* = 0.95 for P; *n* = 15 sessions, Pearson’s correlation, Fig. 2f). We also tested whether the arousal state affected the performance of the monkeys in the detection task. We computed *d*′-sensitivity values by subtracting *z*-scores (normal deviates) of median hit rates from *z*-scores of median false-alarm rates (see Methods). No significant correlations were found between *d*′ values and the pre-cue calls of the corresponding sessions, suggesting that there was no influence of the state of arousal on task performance (*p* = 0.69, *r* = 0.11 for monkey E; *p* = 0.28, *r* = 0.14 for H; *p* = 0.19, *r* = 0.36 for L; *p* = 0.21, *r* = 0.35 for P; *n* = 15 sessions, Pearson’s correlation, Fig. 2f). Our findings show that marmoset monkeys possess the capability to volitionally control vocal output in general.

### Vocal performance during the discrimination task

**Fig. 3:**
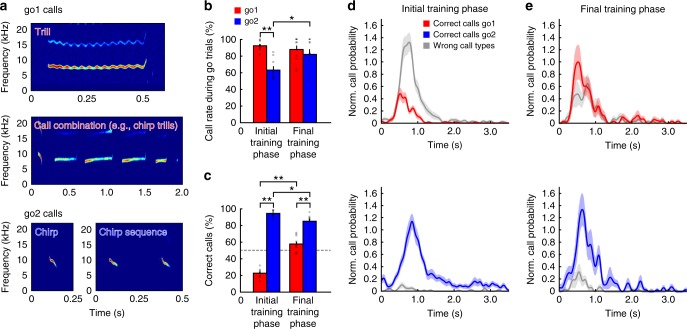
Volitional control of call type in a discrimination task shown in monkey H. **a** Spectrograms of vocalization types. Trills and call combinations had to be uttered in response to the red visual cue (go1); chirps and chirp sequences had to be uttered in response to the blue visual cue (go2). **b** Distribution of go rates for ten sessions in the initial and final training phase of the visual discrimination task. Whiskers indicate standard error (SE). **c** Distribution of correct call types uttered during go1 and go2 cues in the initial and final training phase. Whiskers indicate standard error (SE). **b**, **c** *p < 0.05, **p < 0.01, Wilcoxon signed-rank test. **d**, **e** Call probabilities during go1 and go2 trials in the initial (**d**) and final phase (**e**) of visual discrimination training (**e**). Shaded areas indicate SE

As a next step, we wanted to investigate whether these animals are able to utter different call types on command. We, therefore, trained one of our animals (monkey H) to perform a visual discrimination task (Fig. 1b and Methods for details). Here, the animal had to produce two different types of vocalizations in response to distinct visual cues. As during the visual detection task, the monkey was required to vocalize in response to arbitrary visual cues (red and blue squares). However, here the monkey was trained to utter brief chirp vocalizations or chirp sequences in response to the blue square and to emit long trill calls or call combinations, such as chirp-trill and trill-phee sequences, in response to the red square (Fig. 3a).

In the first 10 sessions after the discrimination task had been introduced (initial training phase), we observed that the animal showed a significantly higher vocal response during the red go-signal (go1) than during the new blue go-signal [go2; 91.9 ± 1.8% vs. 62.8 ± 3.8%; *p* = 2e − 03, *n* = 10 (10 sessions with paired go1 and go2 trial ratios each), Wilcoxon signed-rank test; Fig. 3b]. However, the animal produced significantly more correct call types in the go2 than in the go1 phase (22.7 ± 1.7% vs. 94.1 ± 1.2%; *p* = 2e − 03, *n* = 10, Wilcoxon signed-rank test; Fig. 3c, d). The finding that the monkey showed a higher response to the red go signal might be explained by the use of this signal during the preceding detection task and, therefore, that the animal was still in the state of generalizing to the blue cue as a go-signal in this phase. The better performance during the red go2 signal was consistent with the finding that monkeys predominantly produced chirp vocalizations during the preceding detection task in response to this type of go-stimulus (Fig. 1d). As a result, the yet untrained monkey automatically exhibited a higher probability of uttering a correct rather than wrong call type (Fig. 1b) during the go2 signal and a low probability for uttering a correct call type and a high probability for uttering a wrong call type during the go1 signal (Fig. 3c, d), respectively. We then recorded ten additional sessions after 6 months of training (final training phase). We observed that the monkey significantly increased vocal responses during go2 trials (*p* = 3.71e − 02, *n* = 10, Wilcoxon signed-rank test) to a similar level to its performance during go1 trials (87.4 ± 4.9% vs. 81.5 ± 6.4%; *p* = 0.16, *n* = 10, Wilcoxon signed-rank test; Fig. 3b). Hit rates were still significantly lower during go1 than go2 trials (57.6 ± 3.1% vs. 84.8 ± 2.9%; *p* = 2e − 03, *n* = 10, Wilcoxon signed-rank test; Fig. 3c). In comparison to the initial training phase, hit rates significantly increased for go1 trials (2e − 03, *n* = 10, Wilcoxon signed-rank test), while they slightly yet significantly decreased for go2 trials (1.95e − 02, *n* = 10, Wilcoxon signed-rank test; Fig. 3c). Call rate significantly decreased between the initial and final training phase (*p* = 5.8e − 03, *n* = 10, Wilcoxon rank sum test). Response latencies for correct go1 vocalizations were significantly shorter than for correct go2 vocalization in the initial training phase (median latencies: 646 ms vs. 966 ms; *p* = 1.69e − 21, *n* = 652, Wilcoxon rank sum test; Fig. 3d). In the final training phase, median response latencies for correct go2 calls decreased, resulting in similar response latencies between correct go1 and go2 calls (median latencies: 693 ms vs. 722 ms; *p* = 0.2, *n* = 332, Wilcoxon rank sum test; Fig. 3e).

## Discussion

Our findings demonstrate that marmoset monkeys are capable of volitionally initiating vocal-motor behavior in an operant conditioning task. In contrast to other studies reporting low performance for eye movement or lever pressing tasks^15^, we show that marmoset monkeys can be trained to vocalize on command in response to arbitrary visual cues in a go/no-go detection task. In addition, we report that one marmoset was able to learn to switch between two distinct call types from trial to trial in response to different visual cues in a discrimination task within a few months, indicating that it also has rudimentary control on call type production. When taken together with an earlier study showing that a rhesus monkey can be trained to perform a similar discrimination task^28^, it indicates that the capability to control certain call types might be assumed as a general principle of cognitive vocal behavior in monkeys. Further studies will now have to show that the discrimination results we report on the basis of one marmoset are a general capability of marmoset monkeys. Interestingly, the vocal performance during the discrimination task is lower than during the detection task in both marmoset and rhesus monkeys, indicating distinct constraints in the capability to flexibly control different call types in an operant conditioning task. Nevertheless, our marmoset monkeys used call types of radically different emotional valences, such as tsiks, chirps, and phees, to perform the behavioral tasks^29^. This strongly implies that nonhuman primates (or at least marmosets) are capable of decoupling their vocal utterances from the underlying emotional state to use them independently for successfully performing operant conditioning tasks in a goal-directed way.

Earlier studies showed that juvenile rhesus monkeys are capable of vocal control and producing calls on command in a goal-directed way^21,30,31^. From an evolutionary perspective, our data suggest that the origins of the ability to volitionally control vocal output is much older than previously thought and that the last common ancestor of Old and New World monkeys, which lived more than 35 million years ago^1^, probably had the ability to volitionally control its vocal output. When comparing the vocal performance of these juvenile rhesus monkeys^28^ and the marmoset monkeys of the present study, we observed that the mean number of calls produced during a session (135 calls in macaques vs. 140 calls in marmosets) and mean hit rates (60% vs. 64%) were similar in both monkey species. Differences in vocal performance were found in the mean false alarm rate, which was higher (1% vs. 21%), and mean call response latency during go-stimuli, which was much shorter in marmoset monkeys (1.6 s vs. 0.9 s). We propose that the differences in false alarm rates might be due to the generally higher state of arousal in marmoset monkeys relative to rhesus monkeys, and therefore, a lesser ability of marmosets to inhibit call production than macaques^32^. The higher state of arousal in marmoset monkeys might also lead to a higher degree of “vocal readiness” in the behavioral tasks, which could lead to the observed shorter call response latencies in marmosets. Training marmosets to successfully call on command in a visual detection task takes slightly longer than training macaques in a similar behavioral task^33^ (7 vs. 9 months). However, we show that it is possible to train adult marmoset monkeys to call on command. This is in contrast to a similar macaque study, where it has been reported that juveniles could be trained to reliably vocalize on command but discontinued this controlled vocal behavior in adulthood^34^. This species difference implies that marmosets (like humans) retain a degree of plasticity into adulthood that is greater than in other primates. This makes them a suitable model system to study not only cognitive control mechanisms over a long period per se, but also to investigate developmental and age-related processes that are capable of potentially affecting cognitive motor behavior in primates.

Volitional control of vocal output is a crucial preadaptation for the evolution of human speech in the primate lineage^24,35^. Recent neurophysiological studies in rhesus monkeys found similar activity in brain structures underlying volitional vocal output in monkeys and their homologous structures in the human brain that are crucial for speech production^36,37^. Both structures exhibited similar neural activity related to vocalizations and speech signals, respectively, supporting a continuous evolution of vocal communication systems in the primate lineage ultimately giving rise to speech in humans^24,35^. However, we are just starting to understand the underlying neural mechanisms responsible for the cognitive control of vocal production in primates. We present that marmoset monkeys can be trained to perform complex behavioral tasks, i.e., cognitive control of vocal behavior, in a controlled experimental environment, a prerequisite for being able to pinpoint underlying brain mechanisms. These findings, in combination with the other recently established neurophysiological and genetical tools, make them a suitable primate model to study complex motor behavior in general and the evolutionary aspects of cognitive control underlying human speech in health and disease.

## Methods

### Experimental animals

We used four marmosets (*C. jacchus*; two males, two females) housed at the University of Tübingen, Germany. Animals were usually kept in different sex pairs and were all born in captivity. Monkey H was hand-raised by an animal caretaker from the third postnatal day and reunited with its siblings after 3 months (for details see ref. ^38^). The facility room was maintained at approximately 26 °C, 40–60% relative humidity, and a 12 h:12 h light-dark cycle. They had ad libitum access to water and were fed daily with standard commercial chow and a selection of fruit, vegetables, mealworms, and locusts. Marshmallows and special fruit (e.g., banana and grapes) were used to transfer the animals from their home cages to a transfer box. Experimental procedures were approved by the local authorities of Tübingen (Regierungspräsidium) and were in agreement with the guidelines of the European Community for the care of laboratory animals.

### Data acquisition

Stimulus presentation, behavioral monitoring, and reward presentation were synchronized and performed automatically using a custom-written program (OpenEX and Synapse, Tucker-Davis Technologies, USA) running on a workstation (WS-8 in combination with an RZ2 bioamp processor and RZ6D multi I/O processor, Tucker-Davis Technologies, USA) and a custom-written MATLAB program running on another PC, which was connected via an A/D interface card (PCIe 6321, National Instruments) with the workstation (Fig. 1a). A monitor screen connected to the desktop PC was positioned in front of the animal’s head at a distance of 40 cm for visual stimulus presentation. Vocalizations were recorded using a microphone (MKH 8020 microphone with MZX 8000 preamplifier, Sennheiser, Germany in combination with a phantom power, PAN 48.2, Palmer) positioned 10 cm in front of the monkey’s head and connected to a multi I/O processor (RZ6D, Tucker-Davis Technologies, USA). Vocalizations were recorded using the same system at a sampling rate of 100 kHz. Vocal onset times were detected offline using software (Avisoft-SASLab Pro 5.2.13, Avisoft Bioacoustics) to ensure precise timing for data analysis. The monkey’s behavior was constantly monitored using a USB video camera (Brio, Logitech) placed in front of the monkey.

### Behavioral protocol

The monkeys were trained to sit in a primate chair on their hind legs with their tail in a natural and relaxed position in a soundproof chamber (see Fig. 1a). In the first part of the study, we trained all monkeys to perform a visual go/no-go detection protocol. A trial began when the monkey initiated a ready-response by pushing down on a lever (see Fig. 1a). A visual cue, indicating the no-go-signal (pre-cue; white square, width: 14° of visual angle) appeared for a randomized time from 1 to 5 s for one monkey (monkey H) and 1 to 3 s for the other monkeys (monkey E, L, and P); vocal output had to be withheld during this period. Next, in 80% of trials the visual cue was changed to a colored go-signal (red square; width: 14° of visual angle) lasting for 3000 ms. During this time, the monkey had to emit a vocalization to receive a liquid reward (mixture of water, marshmallows, fruit, marmoset gum, and curd cheese) provided by a small metal syringe directly in front of the monkey’s face. Reward was automatically delivered only when a call was detected during the go-stimulus. In 20% of trials, the pre-cue remained unchanged for another 3000 ms (catch trial). In this period, the monkey had to withhold call production. Catch trials were not rewarded. Calls during catch trials were defined as false alarms. For monkey E and L, we played back audio recordings from the animal facility to maintain their motivational state during the session. One session was recorded per individual per day (5–7 days per week). After habituation to the training setup and initial vocal reinforcement training, the four monkeys were successfully trained to perform the go/no-go detection task with a mean training time of 9 ± 2.5 months.

In the second part of the study, we trained one monkey (monkey H) to perform a visual discrimination protocol, where the animal had to produce two different types of vocalizations in response to distinct visual cues. The other three monkeys were not trained to perform the discrimination task, since they had been directly transferred to other projects in which further training might have been counterproductive. As in during the visual detection task, the monkey initiated a trial by pushing down a lever and the no-go-signal appeared for a randomized time from 1 to 5 s (see Fig. 1b). Next, the visual cue was changed to either a red or blue square. Both go-signals appeared pseudo-randomly with equal probability (*p* = 0.5). Here, the monkey was trained to utter the brief chirp vocalizations, which he predominantly produced during the detection task, in response to the new blue square and long trill calls or call combinations such as chirp-trill and trill-phee sequences in response to the familiar red square. Therefore, the monkey had to learn to produce other types of vocalizations/vocal sequences in response to the known red stimulus and to utter a call type that it predominantly produced in the detection task in response to the new blue stimulus on a trial-by-trial basis to perform the task successfully. The calls that the marmoset was trained to utter in the two conditions are especially distinct and different in their acoustic structure^29,38–41^, and could therefore be easily discriminated by a human observer for reward administration. During the visual discrimination protocol, the monkey had to keep the bar pressed throughout the pre-cue phase to indicate its alertness and bar releases aborted the trial. During the 6 months of visual discrimination training, one session was recorded for this monkey per day (5–7 days per week).

### Data analysis

Fifteen consecutive sessions per individual during the visual detection task and ten consecutive sessions during the visual discrimination task were used in the data analysis. In accordance with the go/no-go detection paradigm, successful go-trials were defined as hits, unsuccessful catch trials as false alarms in the visual detection paradigm. For the visual discrimination protocol, the utterance of the correct vocalization in response to a specific visual cue was defined as a hit a vocal response with the wrong call type as a false alarm.

Since call detection mostly occurred after call offset, call onsets were manually flagged offline using standard software (SASLab Pro version 5.2, Avisoft Bioacoustics, Germany). This procedure ensured the proper timing of call onset for further analyses and prevented the wrong allocation of calls that were uttered directly at the border between two task periods. A recent study reported mean response latencies for a simple motor task, namely saccadic eye movements, of 200 ms in marmoset monkeys^16^. Consequently, we redefined post hoc vocalizations in the first 200 ms following pre-cue onset as calls outside trials, calls in the first 200 ms following go- and catch-trial onset as pre-cue calls, and in the first 200 ms following go- and catch-trial offset as hit and false-alarm calls, respectively. In the current study, we did not aim to classify calls within one call type into subtypes. We classified marmoset vocalizations into previously defined main groups^29,39,42^. Calls were manually classified as chirp, trill, phee, peep, twitter, tsik, or ekk calls based on their spectro-temporal profile and auditory playback. The eight call types show a very defined and distinct profile and could be easily classified manually^29,38–41^. Chirps are calls consisting of a short and descending FM sweep; trill calls are defined by sinusoidal-like FM throughout the call; phee is a tone-like long call with F0 around 7–10 kHz; peeps are short duration tone-like calls that have a sharply ascending or sharply descending FM; twitter is a short upward FM sweep; tsik is a broadband short call consisting of a linearly ascending FM sweep that merges directly into a sharply descending linear FM sweep, and ekk is a short call that is defined as one of the lowest-frequency marmoset calls. Other call types were rarely uttered and defined as “others”. In cases where animals produced call sequences during the vocal detection task, the first call uttered was taken into account for call classification. Call probability distributions were calculated using a moving average (bin width, 500 ms, step size, 1 ms) and smoothed using a Gaussian kernel (bin width, 100 ms; step size, 1 ms) for illustrative purposes only.

### Data normalization

Probability distribution for hit, false alarm, and pre-cue call latencies calculated in the visual detection task were normalized with regard to the hit rate of every single recording session. Probability distributions in the visual discrimination task were normalized for both go-signals with regard to the absolute number of calls uttered within the respective go-trials (go1 and go2) of every single recording session.

### Statistical analysis

Statistical analyses were performed using MATLAB (MathWorks, Natick, MA). We computed *d′* sensitivity values by subtracting *z*-scores (normal deviates) of median hit rates from *z*-scores of median false-alarm rates. Extreme values of hit rates and false-alarm rates were corrected as performed previously^43^. Wilcoxon sign rank tests with Bonferroni correction were calculated to test for significant differences in the vocal performance with respect to the two go-signals (go1 and go2) and the two training phases (initial and final) during the visual discrimination task. We used a three-way ANOVA to test whether animals exhibited different call type ratios at different time points during sessions. Pearson’s correlations were performed to identify relationships between several parameters of vocal behavior. To correct for high sample sizes in the latter test, we introduced the effect size according to Cohen^25^. In all performed tests, significance was tested at an alpha = 0.05 level.

### Reporting summary

Further information on research design is available in the Nature Research Reporting Summary linked to this article.

## Supplementary information

Reporting Summary

## Data Availability

All data needed to evaluate the conclusions in the paper are present in the paper. Additional data related to this paper may be requested from the corresponding author.
