# Author Correction: Cognitive control of complex motor behavior in marmoset monkeys

**DOI:** 10.1038/s41467-019-12531-9

**Published:** 2019-09-26

**Authors:** Thomas Pomberger, Cristina Risueno-Segovia, Yasemin B. Gultekin, Deniz Dohmen, Steffen R. Hage

**Affiliations:** 1 Neurobiology of Vocal Communication, Werner Reichardt Centre for Integrative Neuroscience, University of Tübingen, Otfried-Müller-Str. 25, 72076 Tübingen, Germany; 2 Graduate School of Neural & Behavioural Sciences, International Max Planck Research School, University of Tübingen, Österberg-Str. 3, 72074 Tübingen, Germany

**Keywords:** Psychophysics, Cognitive control, Motor control

Correction to: *Nature Communications*, 10.1038/s41467-019-11714-8, published online 22 August 2019.

The original version of this Article contained an error in the main text, which incorrectly read “(1.6 ms vs. 0.9 ms).” The correct version states “(1.6 s vs. 0.9 s)” in place of “(1.6 ms vs. 0.9 ms).” This has been corrected in both the PDF and HTML versions of the Article.

