# Supplementary material for: Cognitive control of complex motor behavior in marmoset monkeys
Source: Nat Commun. 2019 Aug 22;10:3796. doi: 10.1038/s41467-019-11714-8 (PMC6706403; doi:10.1038/s41467-019-11714-8)
Supplement: Supplementary file 1 — Reporting Summary [file 41467_2019_11714_MOESM1_ESM.pdf]

nature research

Corresponding author(s): Steffen R. Hage

Last updated by author(s): Jul 9, 2019

Reporting Summary

Nature Research wishes to improve the reproducibility of the work that we publish. This form provides structure for consistency and transparency in reporting. For further information on Nature Research policies, see Authors & Referees and the Editorial Policy Checklist.

Statistics

For all statistical analyses, confirm that the following items are present in the figure legend, table legend, main text, or Methods section.

n/a	Confirmed	
☐	☒	The exact sample size (*n*) for each experimental group/condition, given as a discrete number and unit of measurement
☒	☐	A statement on whether measurements were taken from distinct samples or whether the same sample was measured repeatedly
☐	☒	The statistical test(s) used AND whether they are one- or two-sided<br>*Only common tests should be described solely by name; describe more complex techniques in the Methods section.*
☒	☐	A description of all covariates tested
☒	☐	A description of any assumptions or corrections, such as tests of normality and adjustment for multiple comparisons
☐	☒	A full description of the statistical parameters including central tendency (e.g. means) or other basic estimates (e.g. regression coefficient) AND variation (e.g. standard deviation) or associated estimates of uncertainty (e.g. confidence intervals)
☐	☒	For null hypothesis testing, the test statistic (e.g. *F*, *t*, *r*) with confidence intervals, effect sizes, degrees of freedom and *P* value noted<br>*Give P values as exact values whenever suitable.*
☒	☐	For Bayesian analysis, information on the choice of priors and Markov chain Monte Carlo settings
☒	☐	For hierarchical and complex designs, identification of the appropriate level for tests and full reporting of outcomes
☐	☒	Estimates of effect sizes (e.g. Cohen's *d*, Pearson's *r*), indicating how they were calculated

Our web collection on statistics for biologists contains articles on many of the points above.

Software and code

Policy information about availability of computer code

Data collection	Data were collected using Matlab (R2016b, MathWorks, USA), OpenEX and Synapse software (Tucker-Davis Technologies, USA).
Data analysis	Data were analyzed using Matlab (R2016b, MathWorks, USA) and Avisoft (SASLab Pro 5.2.13, Avisoft Bioacoustics, Germany).

For manuscripts utilizing custom algorithms or software that are central to the research but not yet described in published literature, software must be made available to editors/reviewers. We strongly encourage code deposition in a community repository (e.g. GitHub). See the Nature Research guidelines for submitting code & software for further information.

Data

Policy information about availability of data

All manuscripts must include a data availability statement. This statement should provide the following information, where applicable:

- Accession codes, unique identifiers, or web links for publicly available datasets
- A list of figures that have associated raw data
- A description of any restrictions on data availability

All data needed to evaluate the conclusions in the paper are present in the paper. Additional data related to this paper may be requested from the corresponding author.

Field-specific reporting

Please select the one below that is the best fit for your research. If you are not sure, read the appropriate sections before making your selection.

☒ Life sciences ☐ Behavioural & social sciences ☐ Ecological, evolutionary & environmental sciences

Life sciences study design

All studies must disclose on these points even when the disclosure is negative.

Sample size	No sample size calculation was performed. The used sample size (n=4 animals) was well within the range of sample sizes, which have been used in the past in similar studies using demanding operant conditioning tasks in non-human primates (e.g., Hage and Nieder, Nat Commun 2013, n=2; Song et al. 2016, PNAS, n=4; Wasmuht et al. Nat Commun 2018, n=2).
Data exclusions	No data were excluded from data analyses.
Replication	Both operant conditioning tasks being presented in the manuscript have been recorded in a sufficient number of sessions to ensure reproducibility. All attempts of replication were successful.
Randomization	In the first experiment, all animals performed the same behavioral task. In the second experiment, the animal was trained, which was not directly transferred to other projects.
Blinding	Not applicable

Reporting for specific materials, systems and methods

We require information from authors about some types of materials, experimental systems and methods used in many studies. Here, indicate whether each material, system or method listed is relevant to your study. If you are not sure if a list item applies to your research, read the appropriate section before selecting a response.

Materials & experimental systems

n/a	Involved in the study	
☒	☐	Antibodies
☒	☐	Eukaryotic cell lines
☒	☐	Palaeontology
☐	☒	Animals and other organisms
☒	☐	Human research participants
☒	☐	Clinical data

Methods

n/a	Involved in the study	
☒	☐	ChIP-seq
☒	☐	Flow cytometry
☒	☐	MRI-based neuroimaging

Animals and other organisms

Policy information about studies involving animals; ARRIVE guidelines recommended for reporting animal research

Laboratory animals	4 adult Marmoset monkeys (Callithrix jacchus); 2 males, 2 females,
Wild animals	The study did not involve wild animals.
Field-collected samples	The study did not involve samples collected from the field.
Ethics oversight	Experimental procedures were approved by the local authorities of Tübingen (Regierungspräsidium) and were in agreement with the guidelines of the European Community for the care of laboratory animals.

Note that full information on the approval of the study protocol must also be provided in the manuscript.
